# Production of Electrolytic Composite Powder by Nickel Plating of Shredded Polyurethane Foam

**DOI:** 10.3390/ma15113895

**Published:** 2022-05-30

**Authors:** Jolanta Niedbała, Magdalena Popczyk, Łukasz Hawełek, Szymon Orda, Hubert Okła, Jadwiga Gabor, Sebastian Stach, Andrzej S. Swinarew

**Affiliations:** 1 Łukasiewicz Research Network, Institute of Non-Ferrous Metals, Sowińskiego 5, 44-100 Gliwice, Poland; lukasz.hawelek@imn.lukasiewicz.gov.pl (Ł.H.); szymon.orda@imn.lukasiewicz.gov.pl (S.O.); 2 Faculty of Science and Technology, University of Silesia, 75 Pułku Piechoty 1A, 41-500 Chorzów, Poland; hubert.okla@sanepid.gov.pl (H.O.); jadwiga.gabor@us.edu.pl (J.G.); sebastian.stach@us.edu.pl (S.S.); 3 Institute of Sport Science, The Jerzy Kukuczka Academy of Physical Education, Mikołowska 72A, 40-065 Katowice, Poland

**Keywords:** composite powder, amorphous-nanocrystalline nickel, polyurethane foam

## Abstract

Ni–poly(DPU) composite powder was produced under galvanostatic conditions from a nickel bath with the addition of pulverized polymer obtained during the shredding of polyurethane foam (poly(DPU)). The Ni–poly(DPU) composite powder was characterized by the presence of polymer particles covered with an electrolytical amorphous-nanocrystalline nickel coating. The phase structure, chemical composition, morphology, and the distribution of elements was investigated. The chemical analysis showed that the powder contains 41.7% Ni, 16.4% C, 15.7% O, 8.2% P and 0.10% S. The other components were not determined (nitrogen and hydrogen). The phase analysis showed the presence of NiC phase. Composite powder particles are created as a result of the adsorption of Me ions on the fragmented polymer. The current flowing through the galvanic bath forces the flow of the particles. The foam particles with adsorbed nickel ions are transported to the cathode surface, where the Ni^2+^ is discharged. The presence of compound phosphorus in galvanic solution generates the formation of amorphous-nanocrystalline nickel, which covers the polymer particles. The formed nickel–polymer composite powder falls to the bottom of the cell.

## 1. Introduction

Electrochemistry is increasingly used in material engineering in the production of modern and durable materials created by combining various groups of materials. In such materials, interactions at the phase boundary result in a material with modified properties other than the phases from which it was formed [1,2,3,4,5,6,7,8,9,10,11]. In materials of this type, it is possible to obtain a previously unattainable correlation of the phase composition, chemical composition and surface morphology, and thus change the properties of the obtained materials [1,2,3,4,5,6,7,8,9,10,11,12,13,14,15,16].

Metal–polymer composites have enjoyed great interest in recent years [6,7,8,9,10,11,12,13,14,15,16,17,18,19,20,21,22,23]. The smaller the particles, the more interesting the surface properties are, influencing the interfacial properties, agglomeration behavior and physical properties of the particles [7,9,10,11,15,18,21]. Intermolecular interactions in such materials depend mainly on particle surface chemistry, shape, proportion and dimensions, distance between particles and dispersiveness. For the production of metal–polymer composites, a whole spectrum of methods can be used, taking into account the possibility of shaping and molding or replicating polymer-based composites [7,8,9,10,11,12,13,15,16,19,20,23]. The electroless (autocatalytic) method is also used [6,7,13,14,15,16,17,18,19,20]. This method involves the use of a chemical reducing agent to facilitate the reduction of the metal ions in solution to their metallic state. The electrodeposition method is also used to create metal–polymer composites [9,10,11].

The deposition was used, inter alia, for the preparation of composite materials containing metals, such as gold (Au), silver (Ag), copper (Cu) and nickel (Ni) [24,25,26,27]. Alloys were also used. Since composites of this type can be produced in the form of powders, bars, strips, etc., depending on the needs, the choice of method is related to the preferred form of the material. Electroless plating is a low-cost surface metallization process on a variety of polymers. However, this process requires the initial local activation of the inert polymer surface by adsorption of the catalytic particle [11,12,13,14,15,16]. There is no activated group on the surface of the inert polymer, which results in less adhesion between the catalytic particle and the substrate surface. Therefore, it is common to pretreat the polymer surface in several steps, and only after this process is the surface metalized. In recent years, powders of metalized polymers have been the most popular in this group of materials. This is due to the fact that they are heterogeneous systems with pronounced heterogeneity in phase composition, with structures that differ significantly in terms of deformability, thermal resistance and thermal conductivity. These composites offer many benefits such as low cost and corrosion resistance. Potential applications include catalysis, magnetic recording media, materials for protection against electromagnetic interference, and electronic packaging with increased surface area. These materials are also of interest for their use in the design of lightweight shielding systems for radiation protection [6]. In this case, polymer composites are attractive materials because they can be designed to effectively suppress photon or particle radiation. Compared to classic covers, they are much lighter, which significantly improves the comfort of work at workplaces that require their use.

The rapid development of the electronics industry towards miniaturization and high integration has made temperature management of electronic devices also an increasingly important problem [28,29,30,31]. Polymers have excellent mechanical properties and poor thermal conductivity. In order to increase the thermal conductivity of composites dispersed in a polymer matrix, solid fillers with high thermal conductivity are used, such as carbon materials (carbon nanotubes, graphene) [32,33,34], metals (silver, copper) [35,36,37] and ceramics (AlN, BN, Al_2_O_3_) [38,39,40]. Another application of metal–polymer composites is catalysis. Among other things, a composite composed of Ni nanoparticles stabilized with a polymer exhibits catalytic properties. It accelerates the dissolution of carbon dioxide (CO_2_) in brine layers [7]. This material is interesting because of the continuous increase in greenhouse gas emissions attributed to anthropogenic activities. Preferred concentrations of the salt for CO_2_ storage are less than 5% *w*/*v*. Some aquifers have a salinity of up to 25%. Hence, there has been an increased interest in materials that can accelerate the dissolution of CO_2_ to provide a cost-effective method of storing gas in deep saline layers.

Tests of Ni–Mo+X composite materials (X = PPy, PTh and PE) have shown that the presence of polymers in the alloy matrix improves the kinetics of the electrolytic hydrogen evolution process [9,10,11]. Composites are better electrode materials for hydrogen evolution than Ni-Mo alloy. This is probably due to the fact that the introduction of polymers to the alloy matrix causes a significant increase in surface development, including an increase in the electrochemically active surface area in relation to the alloy matrix. Based on the above publications, the authors focused in this work on the electrolytic production of the material—composite powder by nickel plating the fragmented polyurethane foam. The electrolytic technique is often used to obtain various materials because it is cheap and relatively simple, and nickel is widely used as electrode material in electrochemical processes. In the work of [41], the open-cell nickel/polyurethane hybrid metal foams were produced. However, to our knowledge, there is no information about research on nickel-polyurethane composite powder.

Bearing the above in mind, in the presented work, research was carried out to obtain a composite material—metalized polymer powder. Electrolytic nickel was selected as the research material, the selected polymeric additive is powdered duroplastic polyurethane foam (poly(DPU)). Selected physicochemical properties of the obtained powder were characterized and compression tests were carried out.

## 2. Materials and Methods

**Figure 1:**
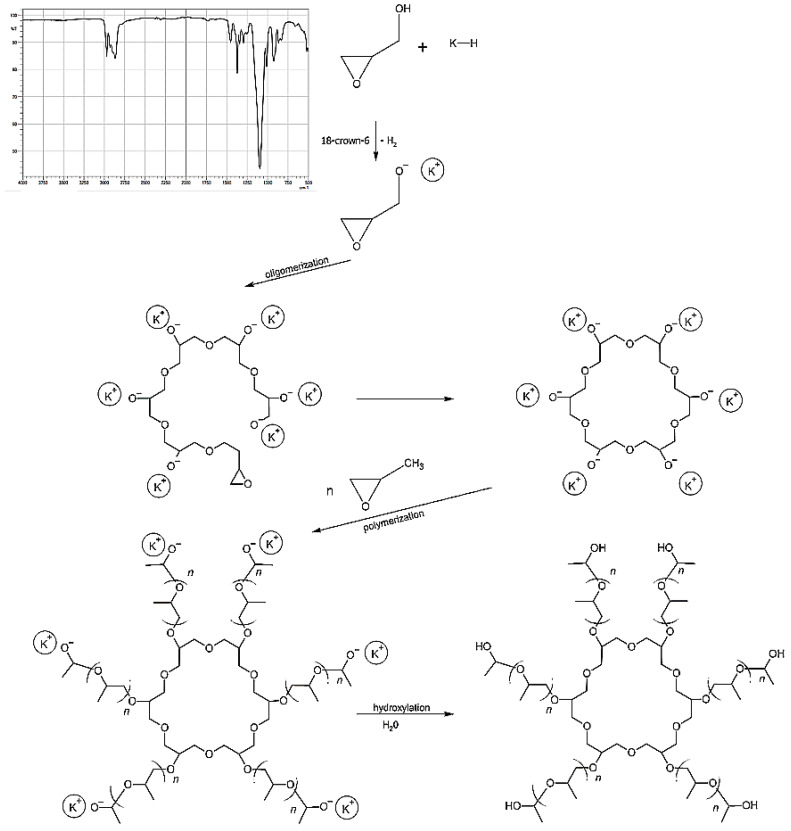
Polyol formation for polyurethane synthesis with FTIR (Fourier-transform infrared spectroscopy) spectra of obtained polyol.

**Figure 2:**
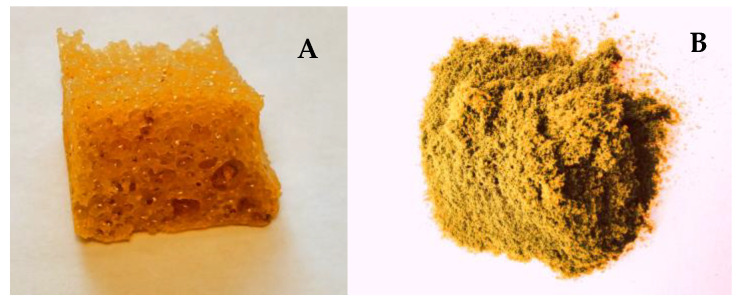
DPU polymer in foam form (**A**) after shredding (**B**).

The first stage of polyurethane preparation was to obtain the base polyol (Figure 1). Poly(DPU) was obtained according to patent number PL 228980 [42], and polyol used for polyurethane synthesis was characterized in the article by Swinarew et al. [43]. The resulting polymeric material is in the form of a light foam (Figure 2A). Before the electrochemical process, the material was shredded. The particles obtained during fragmentation were in the form of fluff (Figure 2B). They were characterized by significant shape variation but did not clump together. The porous structure and developed surface of the material were maintained.

Ni+polymer composite powder was prepared by electrochemical method. A nickel bath modified with the amorphizer NaH_2_PO_2_ (analytical purity, Chempur, Poland) was used. Poly(DPU) polymer powder was added to the bath. A plating bath with the following composition [g/dm^3^] was used: NiSO_4_⋅7H_2_O 55 g, NaH_2_PO_2_⋅H_2_O 58 g, CH_3_COONa 20 g, H_3_BO_3_ 15 g, NH_4_Cl 10 g (all reagents analytical grade, Chempur, Poland). 1 g of shredded polymer was added to the solution. The solution was subjected to “aging” for 24 h with occasional stirring. After this time, the electrolytic process was conducted.

The process of obtaining reduced-density nickel composite was carried out electrochemically on a 0.2 mm thick nickel substrate. The anode was a 0.5 mm thick Ni sheet. Before the deposition process, the cathode surfaces were prepared each time by polishing with sandpaper, degreasing and etching in a hot 1:1 HCl solution (reagents analytical grade, Chempur, Poland). The kit for the composite powder preparation process consisted of a TTi CPX200 DUAL 35 V 10 A PSU, a 1 dm^3^ electrochemical cell. Magnetic and mechanical stirring was used to distribute the polymer particles homogeneously in the bath. Applicable current density 100 mA/cm^2^, pH 4.8–5.5, temperature 55 °C. After the process was complete, stirring was discontinued, and the solution was left until the composite powder was decanted. The non-nickel-coated polymer remained in the upper parts of the bath. The upper portions of the solution were carefully cast to separate the suspension. The residue was filtered, rinsed and dried.

The chemical composition of Ni–poly(DPU) powder was determined by the X-ray fluorescence spectroscopy method using WD XRF spectrometer model ZSX Primus by Rigaku (Osaka, Japan). The radiation source was a lamp operating at 50 kV and an intensity of 50 mA. The LiF200 crystal was used to measure the fluorescent radiation characteristic of the elements from potassium to uranium (according to the position in the periodic table). A germanium crystal was used for fluorescence measurements of carbon, phosphorus, and sulfur, and for the characteristic range of silicon and aluminum, a pentaerythritol crystal was used. Each maximum recorded in the fluorescence intensity spectra from the 2 Θ angle, called the peak or line, was compared with the theoretical angles of 2 Θ tables for the range of analyzed elements. In the case of the theoretical correspondence of this angle with the angle assigned to the line recorded in the spectrum, such a line was marked as a characteristic line for a given element. Oxygen content was determined by the IR method.

Phase composition determination was performed by X-ray diffraction. X-ray diffractometry (XRD) was performed by using a Rigaku MiniFlex600 diffractometer (Rigaku, Tokyo, Japan) equipped with a vertical goniometer (radius of 150 mm), a one-dimensional silicon strip detector D/teX Ultra, CuK_α_ radiation (λ = 1.54 Å) with a Ni filter (for CuK_β_ filtering) operating at a tube voltage of 40 kV and a tube current of 15 mA. The optical setup was in the Bragg–Brentano focusing geometry with a variable divergence slit and a 10 mm receiving slit. Data for the phase identification were recorded in the 2 Θ range from 20 to 90° at 0.02° intervals and a scan speed of 5°/min. The diffraction pattern was analyzed to identify the crystalline phases using the PDXL2 software package.

Surface morphology studies were performed by X-ray microanalysis using JEOL’s JXA 8230 X-ray microprobe (Tokyo, Japan). An accelerating voltage of 15 kV was used. Secondary electron images (SEI, contrast depends mainly on surface topography) and backscattered electron images (COMPO, reveal differences in chemical composition), and elemental distribution maps were made using the energy dispersive method (EDS). The electrolytically obtained Ni–poly(DPU) composite powder was subjected to a pressing process to produce cylindrical pastilles using a Metimex press (Pyskowice, Poland). The process was carried out at room temperature; the applied pressure was 0.3 MPa. After the pressing process, the obtained pastilles were weighed.

Pastilles obtained by pressing with and without conductive resin were analyzed using computer microtomography. Computed microtomography (µCT) is a non-destructive testing method that can map the internal structure of an object from multi-dimensional projections recorded from different angles.

The samples were imaged with a high-resolution X-ray scanner (v|tome|xs, GE Sensing & Inspection Technologies, Phoenix|x-ray, Wunstorf, Germany). First, the test samples were placed in an appropriate rack. Twenty-two hundred projections were performed at 150 kV and 50 μA. The total scan time was 90 min. The determined scanning parameters allowed the registration of images with optimal contrast and 6.5 µm resolution. Projection acquisition was performed using dedicated software (GE Sensing & Inspection Technologies, Wunstorf, Germany). Reconstruction was performed and visualized using VGStudio MAX 2.1 software (Volume Graphics, GmbH., Heidelberg, Germany) in 8-bit grayscale.

A high-resolution X-ray scanner was used to visualize and spatially image the compressed composite powder with and without PVDF. µCT allowed visualization of the microstructure throughout the test sample.

Surface topography studies were performed using LEXT OLS4000 (Olympus, Japan) confocal scanning laser microscope (LCMS), and MountainsMap^®^ Premium software was used for analysis.

## 3. Results

**Figure 3:**
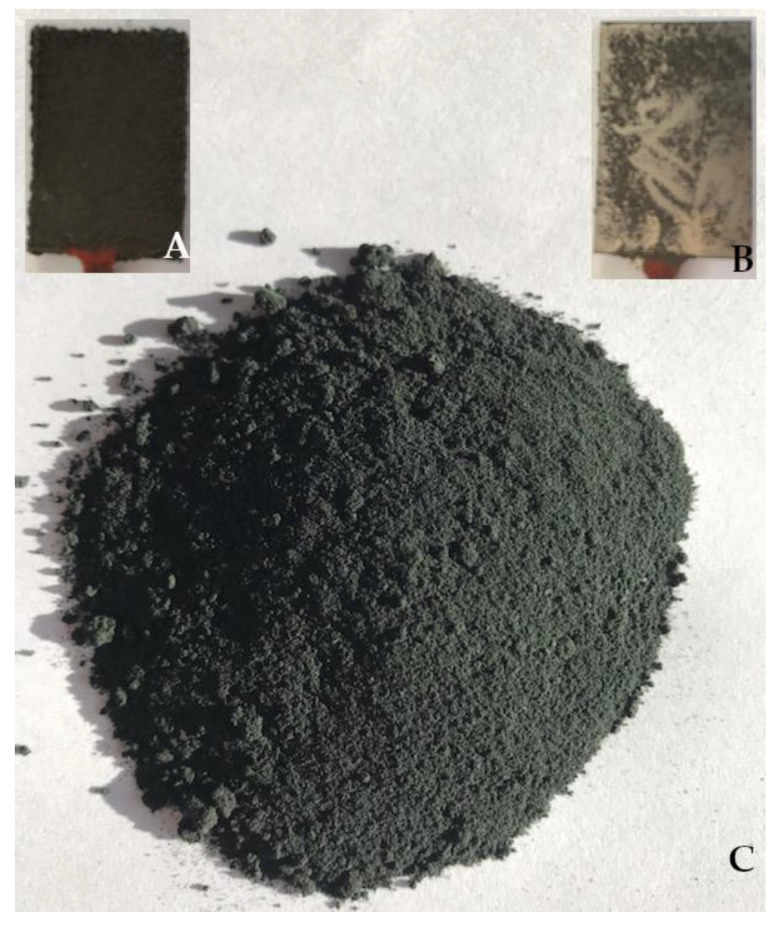
Ni-poly(DPU) composite: (**A**) powder deposited on the cathode surface, (**B**) cathode after powder removal, (**C**) composite powder.

The experiments resulted in a dark-graphite powder built upon the surface of the cathode pad (Figure 3A). During the process, the powder fell steadily to the bottom of the cell. After the process was completed, the material deposited on the cathode surface was easy to separate from the substrate. The surface under the graphite powder was metallic, smooth, and shiny (Figure 3B). Therefore, it can be assumed that the composite formation process begins with the deposition of the metallic nickel coating. This is probably a coating produced from nickel ions that were not adsorbed on the polymer particles and were therefore transported to the cathode surface more quickly. A little later, polymer particles with adsorbed metal ions reach the area near the cathode and are deposited on the formed electrolytic Ni film to form a composite (Figure 3C).

Chemical composition analysis showed that the obtained Ni-poly(DPU) composite powder contained 41.7% Ni, 16.4% C, 15.7% O and 8.2% P (Table 1). The sulfur content was low at 0.10%. In addition to the elements that were determined, the material also includes nitrogen and hydrogen. However, their content was not determined.

**Figure 4:**
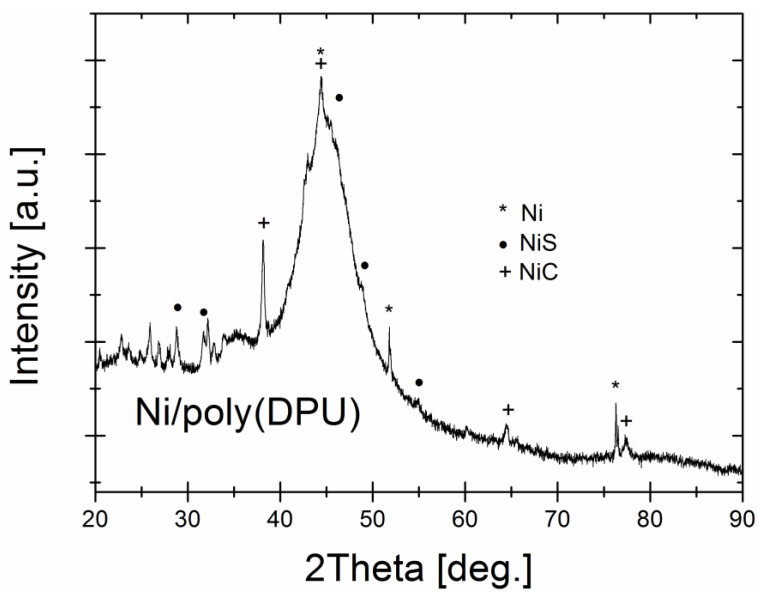
The XRD pattern of the Ni-poly(DPU) composite.

The X-ray diffraction pattern of Ni-poly(DPU) composite is presented in Figure 4. The XRD pattern shows a broad halo region indicating the amorphousness of the Ni nanoparticles with the maximum at 2 Θ = 45 deg and elevated diffused background (the 2 Θ range: 20–35 deg) related with polymer matrix as a dominant composite component, however various sharp Bragg reflections proved the presence of crystalline phases as impurities. Based on recorded reflections, three crystalline phases were identified and fit well with Ni (ICDD (PDF-2) No. 01-078-7533), NiC (ICDD (PDF-2) No. 01-080-3375) and NiS (ICDD (PDF-2) No. 01-074-7239) phases.

**Figure 5:**
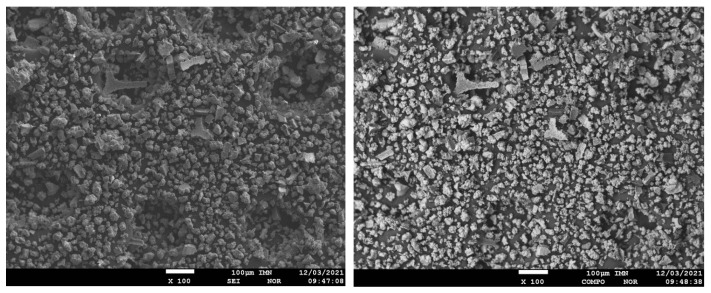

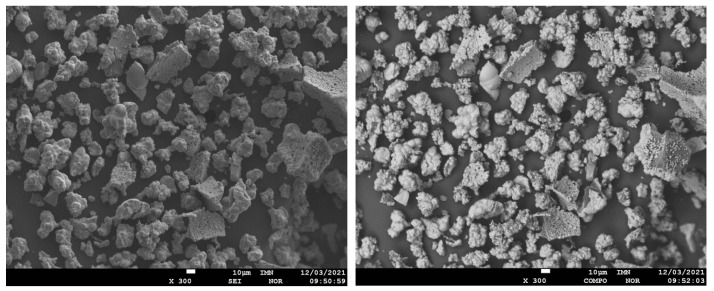
Electron images of Ni-poly(DPU) composite powder sample taken in SEI and COMPO modes, ×100 and ×300 fields.

The surface morphology of the obtained composite powder showed the presence of irregular shapes and a highly developed structure, and significant surface development (Figure 5). The material was characterized by irregular shapes and a three-dimensional structure. This type of morphology was also characteristic of other metallic-polymer materials.

**Figure 6:**
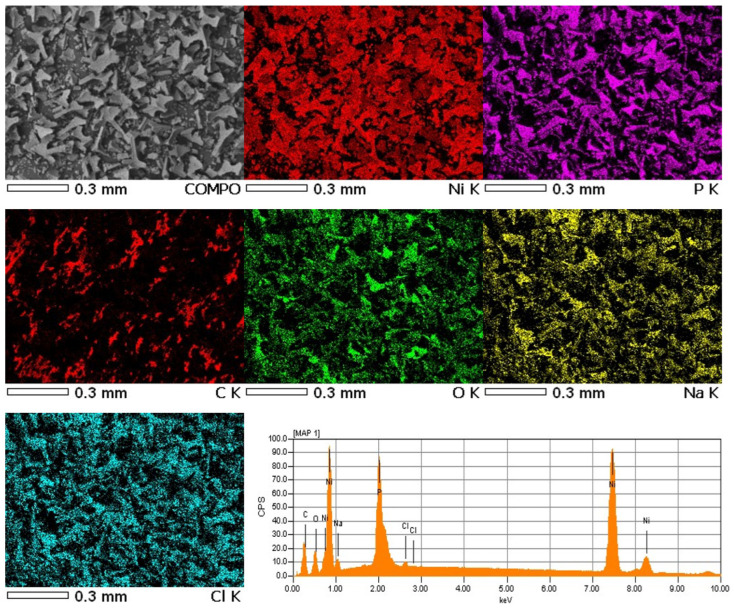
Electron images of a Ni-poly(DPU) composite powder sample along with elemental distribution maps made using the energy dispersive method (EDS).

For the obtained powder, electron images were taken at different magnifications in SEI mode showing its morphology and in COMPO mode showing differences in the chemical composition of individual grains of powder-lighter areas correspond to heavier elements (Figure 5). The samples were applied to a carbon slice to attach the powder grains to the measuring table. Carbon from the slice may slightly interfere with the distribution of carbon from the powder grains (Figure 6). The recorded maps showed an even distribution of elements in the analyzed Ni-poly(DPU) powder. In addition to the elements in the composite, the presence of a small amount of sodium was recorded. This is likely due to inaccurate washing of the sample.

**Figure 7:**
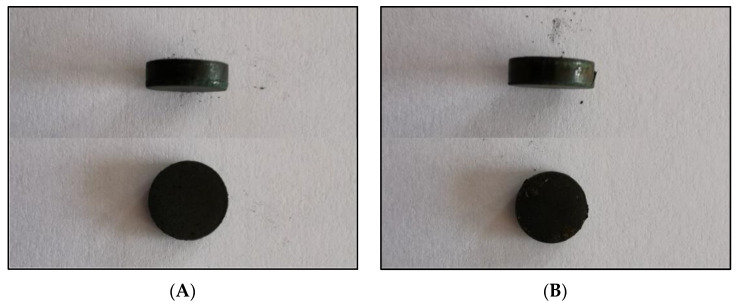
Compressed electrolytic Ni–poly(DPU) composite powder; (**A**) compressed material without PVDF, (**B**) pastille obtained by compression molding with PVDF ((polyvinylidene fluoride) conductive resin).

Amorphous-nanocrystalline Ni-poly(DPU) was subjected to a pressing process. Since the durability of the formed pastille could not be predicted in advance, pressing was carried out not only of the material itself, but also of the material with an additive of conductive resin (PVDF; polyvinylidene fluoride). The material obtained both without and with the addition of resin was very easy to press (Figure 7). The material obtained from pressing both with and without resin is well integrated, homogeneous, and does not crumble or crack. Dimensions of the resulting pastilles: diameter 10 mm, h = 3 mm (Figure 7). The mass of the pastille made by embedding the composite powder in conductive resin (PVDF) (polyvinylidene fluoride) and pressing is M = 513.09 mg, including M powder = 502.48 mg, and M resin = 10.61 mg. For the pastille without resin addition mass was M = 507.77 mg. The volume of both is 0.2335 cm^3^, and the calculated density of the material 2.1558 g/cm^3^. The mass of Ni pastille of the same size is M = 2.08 g, density 8.908 g/cm^3^, respectively. Thus, the analyzed composite material has almost four times lower density (3.75×) compared to Ni pastil without polymer addition.

**Figure 8:**
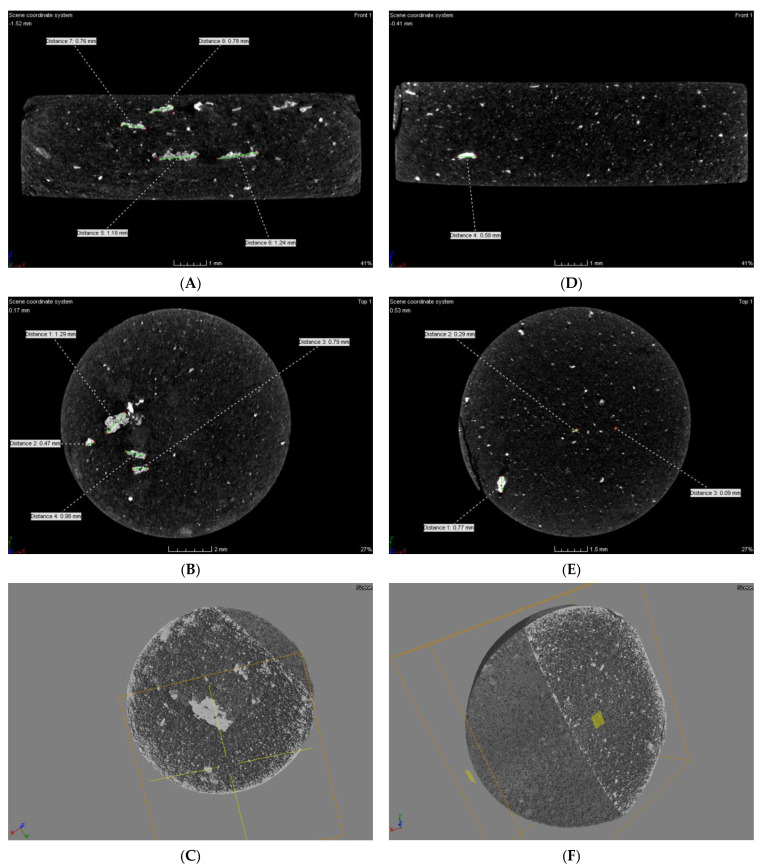
Microtomographic analysis image of compressed composite material: (**A**–**C**) Ni–poly(DPU); (**D**–**F**) Ni–poly(DPU) powder with PVDF resin addition.

Measurement of the linear X-ray absorption coefficient allows differentiation of structures in the material. Each structure has its own x-ray attenuation factor, and the µCT image is described in relation to it. The measurement criterion to determine the occurrence of hypodense, hyperdense and isodense areas are Hounsfield Units (HU), which define radiographic density. These areas are delineated by evaluating the density of X-rays (X-rays) used during the examination. Areas with an increased absorption coefficient of X-rays relative to the surroundings (bright) are called hyperdense, and those with a lower absorption coefficient (dark) are called hypodense. Structures that are indistinguishable from their surroundings are isodense. The tomographic analysis of the obtained material in the microstructure of both pastilles, without and with the addition of the conductive resin, showed the presence of both hypo- and hyperdense areas. In the compressed materials, a uniform distribution of nickel clusters was observed in the cross-sections of the tablets made of the modified polymer with PVDF. The size of agglomerates, in this case, was in the range of 0.09–0.77 mm. For the material without PVDF additive, the size of metal agglomerates was in the range of 0.47–1.29 mm (Figure 8A–C).

The compressed material of both the composite alone (Figure 8A–C) and the composite with resin (Figure 8D–F) was characterized by a relatively uniform filling across the volume. From the images obtained on the cross-section and oblique section, it could be seen that in the case of the compressed material without conductive resin and with resin, the material was characterized by a porous, pumice-like structure and high surface development throughout the volume. The structure was very uniform. No gas occlusions, craters, or internal cracks were observed in the material. Both materials are homogeneous throughout.

**Figure 9:**
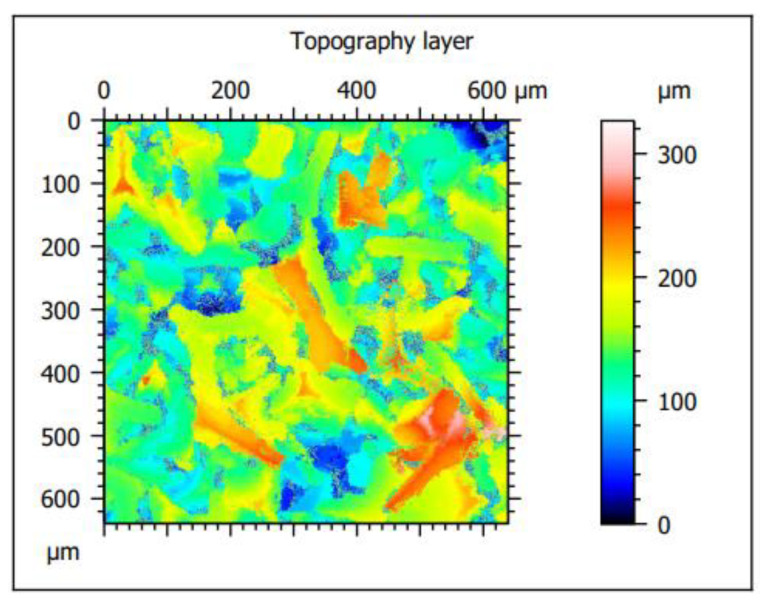

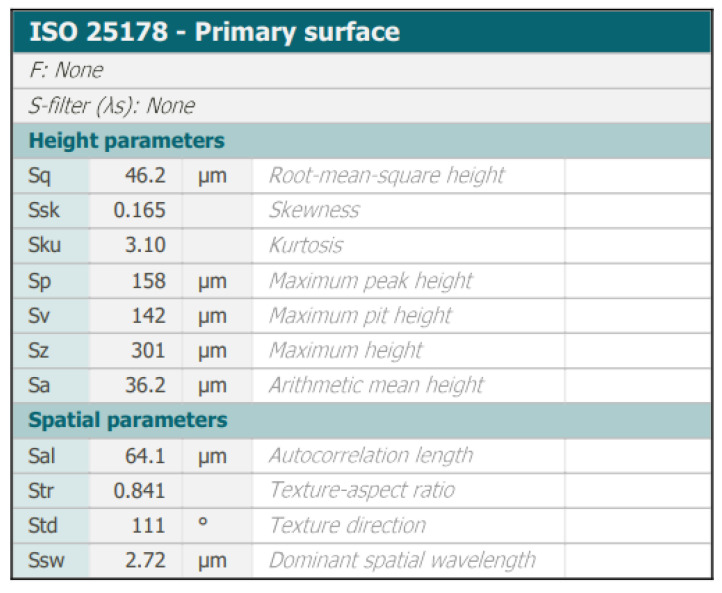
Confocal microscope analysis—2D layer topography and stereometric parameters obtained according to the ISO 25178 standard.

**Figure 10:**
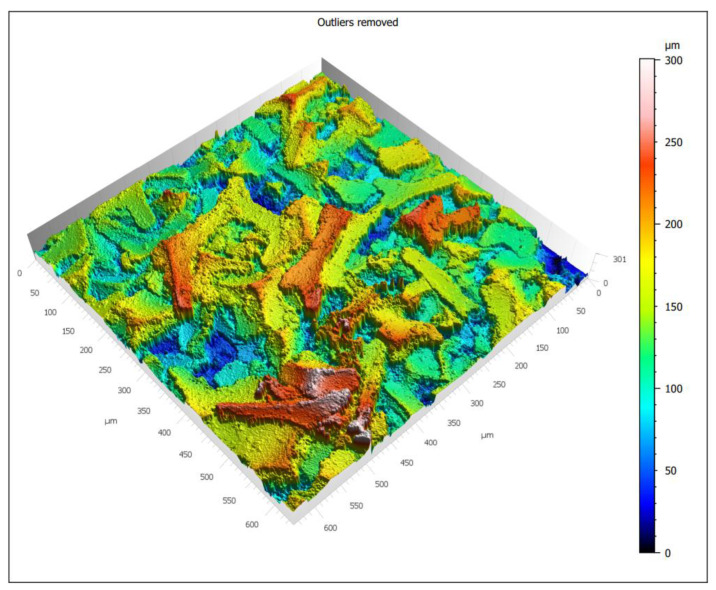
3D Confocal miscroscope micrograps.

The analyzed material was characterized by the presence of microstructures with a significant difference in particle size and a very well-developed surface (Figure 9 and Figure 10). The microscopic image clearly shows polymer particles of various sizes. On their entire surface, regardless of the size and shape of the polymer particle, regular, small grains of the Ni-P alloy were visible. The obtained images confirm the XRD analyses presented earlier, which showed the presence of amorphous Ni on polymer particles. The complex nature of the surface made it rough and highly developed. Such a structure of the composite—polymer particles with amorphous-nanocrystalline nickel grains present—gives a material with a significant active surface. The summary of the obtained results of microtomographic, confocal and XRD tests allows concluding that this material, due to the nature of the surface, can be used as a material for electro-emission or electro-storage of gases (e.g., hydrogen).

## 4. Discussion

Thanks to the combination of two materials (metal/polymer) with significantly different properties in the obtained composite powder, a new material was created that differs in morphology, topography and surface development compared to the base materials. The analysis of the results of electrolytic forming of the powder–polymer composite shows that it is a two-stage process. The first stage is related to the formation of the Ni-P coating on the surface of the fragmented polymer particles. In the second stage, polymer particles with adsorbed metal ions are deposited on the resulting metal coating, creating a composite.

The analysis of the chemical composition of the obtained material showed the presence of about 42 wt.% Ni and a little over 8 wt.% P. The phosphorus content in the range of 5–9 wt.% is characteristic of the amorphous-nanocrystalline Ni-P structure [44].

The composite powder consists of approximately 50% nanocrystalline nickel and 50% polyurethane foam. The obtained proportions confirm that the applied electrochemical method is suitable for the production of this type of composite. The work to date [9,10,11] allowed to obtain metal–polymer composites containing about 3–35% polymers with the electrochemical method. The experiments were performed with conductive and non-conductive polymers. The obtained result is also confirmed by the use of appropriate current density values from the material production process. Since the preparation is a cathode process, at too high current densities, intensive hydrogen evolution occurs, which makes it difficult for dispersed particles to access the cathode surface, which limits the process of composite powder formation by controlling the diffusion of the process. A similar phenomenon is observed in the case of electrodeposition of composite coatings with metal powders and metal oxides or semiconductors [45,46,47]. Therefore, it can be assumed that the mechanism of formation of these composite materials is similar. As the presented test results show, the advantage of the obtained composite powder is the possibility of pressing without the use of resin, which limits the number of components introduced into the material, and thus, facilitates its characterization when used as an electrode material.

Computed microtomography allowed to obtaining an accurate, three-dimensional image of the microstructure of the tested material thanks to the differences in the absorption properties of materials, which, depending on their composition, absorb electromagnetic radiation to a different extent [48].

Confocal microscopy shows that the obtained structure and surface topography are characteristic of metal–polymer materials. An example may be Ni (II)-IIP material obtained by bulk polymerization [12]. Like the tested composite powder, it is characterized by non-circular shapes and a diversified three-dimensional structure.

## 5. Conclusions

The electrochemical method allows the production of Ni–poly(DPU) composite powder with a reduced density compared to Ni powder. The obtained material contains 41.7% Ni, 16.4% C, 15.7% O, and 8.2% P, 0.10% S. The other components are nitrogen and hydrogen.

The phase analysis showed that the Ni–poly(DPU) powder is characterized by an amorphous-nanocrystalline structure, the recorded dominant phase being NiC.

Surface morphology studies revealed the complex nature of the material structure. The microscopic image shows fragmented polymer particles coated with nickel material.

The composite powder is very easy to press and has a porous, pumice-like structure, and is characterized by high surface development. No gas occlusions, craters, or internal cracks were observed in the material. The obtained composite powder (Ni–poly(DPU)) after pressing can be used as electrode material in various electrochemical processes.

The proposed method of obtaining Ni–poly(DPU) composite powder allows the use of polymer waste (for example, with recycling of polyurethane foams). In this case, modern material with a developed surface was produced from polymer waste.

## Figures and Tables

**Table 1:** Chemical composition of Ni–poly(DPU) composite powder.

Element	% Ni	% C	% O	% P	% S
Content (weight %)	41.7	16.4	15.7	8.2	0.10

## Data Availability

All data are contained within the article.
